# Acute Pretreatment with Chloroquine Attenuates Renal I/R Injury in Rats

**DOI:** 10.1371/journal.pone.0092673

**Published:** 2014-03-28

**Authors:** Zoran Todorovic, Branislava Medic, Gordana Basta-Jovanovic, Sanja Radojevic Skodric, Radan Stojanovic, Branislav Rovcanin, Milica Prostran

**Affiliations:** 1 Department of Pharmacology, Clinical Pharmacology and Toxicology, Faculty of Medicine, University of Belgrade, Belgrade, Serbia; 2 Department of Pathology, Faculty of Medicine, University of Belgrade, Belgrade, Serbia; 3 Department of Human Genetics, Faculty of Medicine, University of Belgrade, Belgrade, Serbia; The University of Manchester, United Kingdom

## Abstract

**Background:**

Acute kidney injury (AKI) still remains an unresolved problem in pharmacotherapy and renal inflammation is a major factor in its development. Chloroquine, a well-known antimalarial drug, posses pleitropic effects as well: antiinflammatory, anticoagulant and vascular actions. The effects of chloroquine on renal function may involve significant increase in urine flow rate, glomerular filtration rate and sodium excretion, as well as stimulation of nitric oxide synthase. However, its role in experimental models of renal I/R injury is unknown. We aimed to analyze the acute effects of a single-dose intravenous chloroquine administered at three different times in the experimental model of I/R injury in rat.

**Methods:**

Rats were subjected to bilateral renal ischemia (45 min) followed by reperfusion with saline lasting 4 hours. Chloroquine was administered in doses of 0.3 mg/kg i.v. and 3 mg/kg i.v. 30 min before ischemia, 30 min before reperfusion and 5 min before reperfusion. Selected a hemodynamic, biochemical and morphological parameters were followed in the Sham-operated animals and rats subjected to I/R injury and pretreated with saline or chloroquine.

**Results:**

Chloroquine (0.3 and 3 mg/kg, i.v.) protected the I/R injured kidney in an U-shaped manner. Both doses were protective regarding biochemical and histological markers of the I/R injury (serum urea, creatinine and fractional excretion of sodium, as well as total histological score, tubular necrosis score and KIM-1 staining score) (P<0.05 vs. corresponding controls, i.e. rats subjected to I/R injury and treated with saline only). The protective effects of the lower dose of chloroquine were more profound. Time-related differences between pretreatments were not observed (P>0.05, all).

**Conclusion:**

Our study shows for the first time that a single dose of chloroquine (0.3 mg/kg i.v.) could afford significant protection of the injured rat kidney.

## Introduction

Acute kidney injury (AKI), previously referred to as acute renal failure (ARF), is a heterogeneous process, that is usually classified into three different groups: (a) prerenal, which is an adaptive mechanism of severe renal damage (hypovolemia and/or hypotension), (b) intrinsic, which is structural renal injury and (c) postrenal, which represents mechanical obstruction of the urinary system. Renal ischemia-reperfusion (I/R) injury is one of the most common causes of prerenal AKI [1].

Despite novel therapeutic strategies, I/R injury remains an unresolved problem in pharmacotherapy with high mortality rate, especially in critically ill patients. Hospital mortality has not decreased significantly over last sixty years and it varies from 28% to 90% [2].

The important role of inflammation in the development of renal ischemia-reperfusion injury is confirmed in many studies [3], [4]. Some clinical and a lot of experimental data suggest that I/R refers to an complex inflamatory process that includes leukocyte trafficking, synthesis of pro-inflammatory cytokines such as interleukin (IL)-1, IL-6 and tumor necrosis factor (TNF)-α, increasing in cell adhesion-molecule expression, development of oxidative stress, changes in permeability of membrane ion and water-channel expression, dysfunction in calcium homeostasis, phospholipase and protease activation etc. [5], [6].

Mainly, oxidative stress in the kidney is caused by polymorfonuclear (PMN) and macrophages infiltration and production of reactive oxygen species (ROS) and reactive nitrogen species. On the other hand, there is an evidence that some NO-donor at low concentration can reduce inflammation during I/R injury [7].

Also, different authors have recently confirmed that autophagy is induced in renal tubular cells during AKI [8].

Some increasing evidence suggests that antiinflammatory and antimalarial drug, chloroquine, which is also a pharmacological inhibitor of autophagy, may influence renal function by stimulating a significant increase in urine flow rate, glomerular filtration rate and sodium excretion. The effects of chloroquine on renal function probably involves stimulation of nitric oxide synthase [9]. Despite that, chloroquine has a controversial role on the expression of iNOS, a property that is responsible for many of physiological effects in organs such as the kidneys. It seems that effects of chloroquine on NO production are cell-type dependent. Some previous studies showed inhibition of iNOS activity and NO synthesis in a dose-dependent manner in a murine peritoneal macrophages that have been stimulated with IFN-γ [10]. Also, it modulates both glomerular hemodynamics and tubular function [11].

Preventive strategies are most effective when started before oliguria and elevated serum creatinine which are delayed and unreliable markers of kidney dammage. One of new promising, early biomarker is kidney injury molecule 1 (KIM-1) that is believed to participate in the regeneration process. KIM-1 is an important biomarker of AKI and acute tubular necrosis (ATN) and correlates with the degree of renal dysfunction. The KIM-1 gene (or protein) expression is undetectable in normal kidney, but this molecule is usually present in damaged tubular epithelial cells. Also, some authors detected significant increase of urinary KIM-1 in rat experimental model of I/R renal injury, without any changes in the level of plasma creatinine, creatinine clearance or proteinuria. A rapid testing method for KIM-1 has been described, yielding semiquantitative results in just 15 minutes [12], [13]. One prospective study was shown that KIM-1 can even predict adverse clinical outcomes in patients with AKI: patients with the highest levels in urinary KIM-1 had the highest odds for dialysis and hospital death [14]–[16].

The aim of our study was to analyze the acute effects of a single-dose intravenous chloroquine administered at three different times in the experimental model of I/R in rat. The analysis would take into account selected biochemical and histological markers of glomerular and tubular function and reperfusion injury, as well as semiquantitative scoring of KIM-1 staining.

## Materials and Methods

### Methods are desribed in detail elsewhere [14], [17]–[19]. The experimental design is shown in Fig.1

In brief, *in vivo* experiments were performed in male adult Wistar rats (N = 66) weighing 200–300 g (239±11 g) and receiving a standard diet and water *ad libitum*. This study was carried out in strict accordance with the Animal Welfare Act of the Republic of Serbia (Official Gazette of the Republic of Serbia No. 41/09), Directive 2010/63/EU on the Protection of Animals Used for Scientific Purposes, and the Guide for the Care and Use of Laboratory Animals (National Research Council, 8^th^ ed., USA). The protocol was approved by the Committee on the Ethics of Animal Experiments of the Faculty of Medicine, University of Belgrade (License Number: 2394/2).

**Figure 1:**
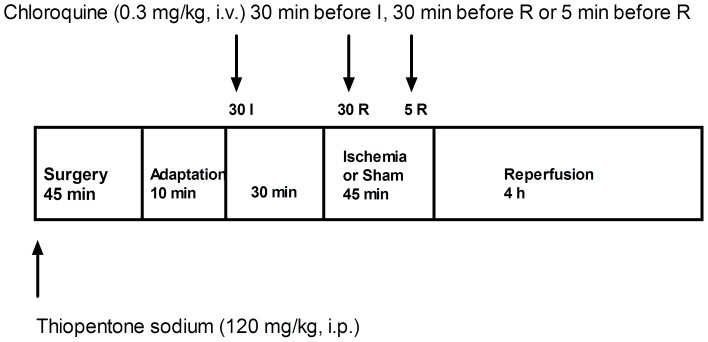
Experimental design. Chloroquine was injected 30(30I-, 30R- and 5R-group, respectively).

The following compounds were used: chloroquine diphosphate salt (Sigma-Aldrich, Poole, Dorset, UK), sodium thiopentone (Thiopental®, Nycomed Pharma, Unterschleibheim, Germany) and nonpyrogenic saline 0.9% w/v NaCl (Hemofarm, Vršac, Serbia).

Male adult Wistar rats are anesthezied with intraperitoneal bolus injection of sodium thiopentone (120 mg/kg) and placed on their backs on thermostatically controlled heating mat to provide constant body temperature 37.5±1C. Trachea, carotid artery, jugular vein and urinary bladder are separated from surrounding tissue and inserted with appropriate tubes or cannulas. Afterwards, both renal pedicles are isolated from the surrounding tissue, separated from ureter, and clamped with appropriate artery clip to reversibly stop blood supply to and from kidneys. Ischemia (pedicles are clipped) lasted 45 min. Clips are removed from pedicles with big forceps at the beginning of reperfusion, which lasted 4 h. Saline infusion is provided during the whole experiment (8 ml/kg/h before reperfusion and 2 ml/kg/h during reperfusion). In addition, cardiovascular parameters are monitored continuosly, and anesthesia is maintained by supplementary injections of thiopentone sodium (10 mg/kg, i.v.), as required All animals received a continuous infusion of 0.9% (w/v) saline 8 ml/kg/h, i.v. during a period of adaptation (30 min) and during ischemia (45 min), and 2 ml/kg/h during reperfusion time of 4 h. At the end of all experiments, rats were euthanized using an overdose of sodium thiopentone [14].

At the beginning, chloroquine screening experiments were performed (N = 2–4 per group). Chloroquine was administered in doses of 0.3 mg/kg i.v., 3 mg/kg i.v. and 30 mg/kg i.v. 30 min before ischemia, 30 min before reperfusion and 5 min before reperfusion. Time-related differences between pretreatment were not observed in either of groups. A dose of 30 mg/kg of chloroquine caused a marked decrease in the mean arterial pressure during the i.v. application, so in this paper we will only discuss the effects of the lower doses.

Rats were randomized into six experimental groups (N = 6–10 per group):

- I: Sham-operated + saline (0.9% NaCl),
- II: IR + saline,
- III: IR + chloroquine 0.3 mg/kg, 30 min before ischemia (IR + H03-30I),
- IV: IR + chloroquine 0.3 mg/kg, 30 min before reperfusion (IR + H03-30R),
- V: IR + chloroquine 0.3 mg/kg, 5 min before reperfusion (IR + H03-5R),
- VI: IR + chloroquine 3 mg/kg, 30 min before ischemia (IR + H3-30I).

In all groups during reperfusion urine was collected, and after the end of the experiment, blood samples were taken and analyzed for markers of renal impairment (sUr, SCre, FENa^+^).

Fractional excretion of sodium (FENa+) is calculated using standard formula incorporating the ratio of serum and urinary sodium concentrations (sNa and uNa), clearance of creatinine (Ccl) and urine flow [14].

Also, *post mortem* samples of both kidneys of each animal were taken for histological evaluation. The samples of kidney were placed in formalin and processed through to wax, subsequently sectioned at 5 μm and stained with PAS (Periodic acid-Schiff). Original magnification x 20 was used. Each figure shown was randomly chosen from the series of at least 6 experiments (electronic light microscope type Leica DM LS 2, type 11020518016, Microsystems, Wetzlar, Germany). The kidney samples were then graded histologically according to the severity of injury by using a predetermined scoring system [20]. The histological score evaluated were tubular necrosis, interstitial edema, loss of brush border and casts formation. A minimum of 10 fields for each kidney slide were examined and assigned for severity of changes. The scoring system used was 0, absent; 1, minimal changes, 2, moderate changes and 3, marked changes. Total score per kidneys was calculated by addition of all scores. It should be pointed out that blinded analysis of the histological samples was performed by two experts (Department of Pathology, Faculty of Medicine, University of Belgrade).

### Immunohistochemistry

Mouse monoclonal antibody to KIM-1 (MAB1750) was purchased by R&D systems Europe (Abingdon, UK). Sections (5-μ thick) from the formalin-fixed, paraffin-embedded tissue samples were deparaffinized and treated with 3% hydrogen peroxide for 15 min to block endogenous peroxidase activity. For the heat-induced antigen retrieval, tissue sections were immersed in 0.01 mol/ L citrate buffer (pH = 6.0) and treated in a microwave oven for 20 min at 620 W. After cooling off for 30 min at room temperature, blocking peptide (DAKO, Glostrup, Denmark) was utilized to block the non-specific staining and primary antibody, diluted 1∶20, was applied overnight, at 4°C. Streptavidin-biotin technique using DAKO's LSAB+ kit (DAKO, Denmark) was applied, with diaminobenzidine (DAB) as the chromogen solution and Mayer's hematoxylin for the counterstain. Normal epithelial tissue was included in every staining procedure as a positive control for KIM-1, whereas incubation with the pure antibody diluent (without the primary antibody) served as a negative control. During the development of immunohistochemical protocol for KIM-1 detection, we performed immunostaining with monoclonal and polyclonal antibodies. As positive control we used testicular tissue, considering the fact that abundant KIM-1 expression can be detect in kidney and testis with application of polyclonal anti KIM-1 antibody. For negative control, we left out primary antibody during immunostaining procedure.

As we obtained identical results using monoclonal and polyclonal antibodies in several analyzed samples as well as in positive and negative control, we carried on our experiment with monoclonal antibody.

Similarity between human and rat extracellular domain of Kim-1 protein, was determined by comparison of amino acid sequences. Using the UniProt database, amino acid sequences of human and rat Kim-1 were accessed by following entries Q96D42 and O54947, respectively. Sequence alignment was performed by using BLAST tool.

These domains show 57% of sequence identity (E-value 5e-41) and most of differences are due to the existence of repeated elements in human EC domain of Kim-1. The Ig like V subdomain of EC domain is characterized by 57% (E-value 2e-39). Similarity between human and rat Kim-1 EC domain suggests that structural conditions are fulfilled for equalization of ligand binding and overall biochemical similarity. The sequence conservation in both species suggests the significant selective pressure against sequence alteration during evolution of Kim-1 gene. The human anti-EC Kim-1 Mab binds to the same domain of rat's Kim-1, leading to a conclusion that experimental results based on rat's model could be seriously taken into concern for extrapolation to humans [21], [22].

Localisation of immunohistochemical staining was assessed by light microscopy. Brown-coloured products in the cytoplasm identified positive staining. The results of immunohistochemical staining were scored by semiquatitive technique: absence of staining in all epithelial cells (negative staining); positive staining involving less than 10% of cells (focal expression; +), 10%–50% positive cells (moderate expression), and more than 50% positive cells (diffuse expression).

Kidney injury molecule-1 positive staining intensity in proximal tubules of all samples was evaluated manually. The staining intensity score of targeted and control epithelial cells was graded from 0 to 3 according to scoring system described by Zang et al. [23].

The KIM-1 staining intensity score for each sample was graded by two experts in the field of pathology who was blinded to the group assessments.

### Statistical analysis

All values described in the text and figures are expressed as the mean ± standard error of the mean (S.E.M.) of n observations. For *in vivo* studies, each data point represents biochemical measurements or histological scores obrained from 6–10 separate animals. Statistical analysis was carried out using one-way analysis of variance (ANOVA) followed by *Bonferroni's* post-hoc test. Correlation between KIM-1 staining score and other results and scores was assessed using nonparametric Spearman test. All mentioned statistical analysis was performed by using GraphPad Prism/Instat 1.1 (GraphPad Software, San Diego, CA, USA). A *P* value of less than 0.05 was considered significant (NS = *P*>0.05 =  non significant).

## Results

Neither saline nor chloroquine at doses of 0.3 mg/kg and 3 mg/kg affected the mean arterial pressure (MAP) and heart rate (HR) of anesthetized rats (not shown). On the other hand, chloroquine in dose of 30 mg/kg led to a major decrease in MAP, without significant changes in HR. In rats subjected to I/R injury, renal artery occlusion per se caused a transient fall in MAP and HR.

**Figure 2:**
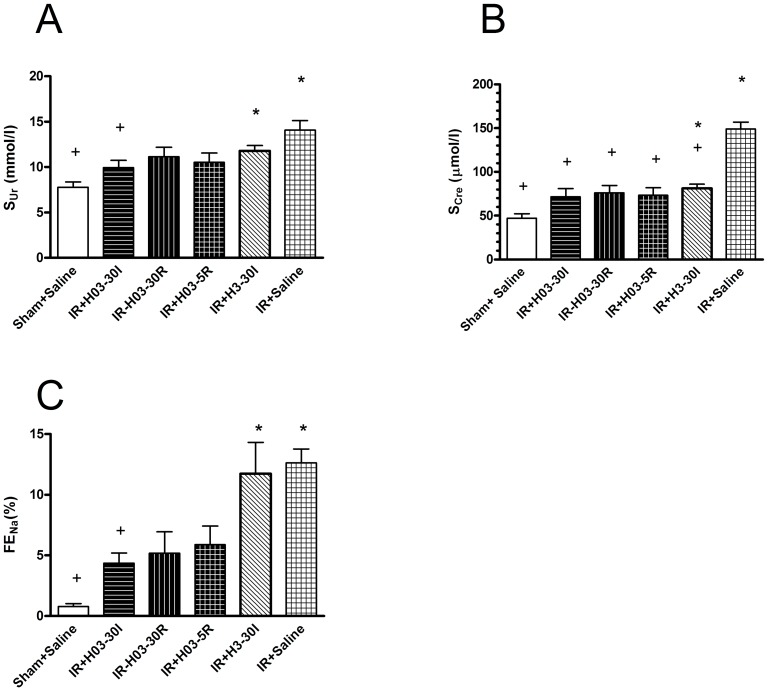
The effects of chloroquine (0.3 mg/kg, i.v; 3 mg/kg, i.v.) on renal dysfunction and injury caused by I/R. Chloroquine in dose of 0.3/kg, i.v. was injected 30 min before ischemia (IR+H03-30I), 30 min before reperfusion (IR+ H03-30R) and 5 min before reperfusion (IR+H03-5R), and in dose of 3 mg/kg, i.v. was injected 30 min before ischemia (IR+H3-30I). Control groups, Sham+Saline and IR+ Saline received an i.v. bolus of 0.5 ml saline only. Panel A, B, and C: serum creatinine and urea concentrations and fractional excretion of Na^+^. Each bar represants mean ± S.E.M. +: P<0.05 *vs*. IR+Saline, *: P<0.05 *vs*.Sham+Saline (N = 6–10 rats).

I/R injury significantly increased serum urea and creatinine concentrations (sUr, sCre) and fractional excretion of sodium (FENa^+^) (IR + Saline *vs*. Sham + Saline; P<0.01) (Fig. 2A–C)

Chloroquine (0.3 mg/kg i.v) significantly reduced sCre regardless of the time of injection in comparasion with control group (IR + H03-30I, IR + H03-30R and IR + H03-5R *vs*. IR + Saline; P<0.01). However, chloroquine in dose of 3 mg/kg, i.v. reduced, but did not completely abolished the effects of I/R injury (IR + H3-30I vs. IR + Saline; P<0.05) (Fig. 2A)

Also, chloroquine in the lowest dose reduced sUr and FENa^+^ in comparasion with control group (IR + H03-30I vs. IR + Saline; P<0.05). Time-related differences between pretreatments were not observed (IR + H03-30I and IR + H03-30R *vs*. IR + H03-5R; P>0.05, all) (Fig. 2B, C).

**Figure 3:**
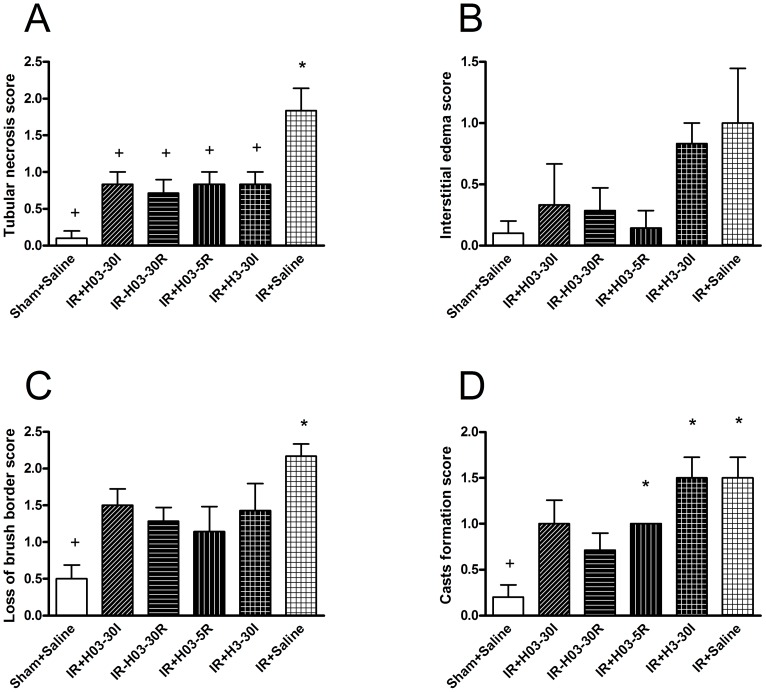
The effects of chloroquine (0.3 mg/kg; 3 mg/kg, i.v. bolus) on histological score of renal I/R injury. Chloroquine in dose of 0.3/kg, i.v. was injected 30 min before ischemia (IR+H03-30I), 30 min before reperfusion (IR+ H03-30R) and 5 min before reperfusion (IR+H03-5R), and in dose of 3 mg/kg, i.v. was injected 30 min before ischemia (IR+H3-30I). Control groups, Sham+Saline and IR+ Saline received an i.v. bolus of 0.5 ml saline only. The histological parameters evaluated were tubular necrosis, interstitial edema, loss of brush border, and cast formation score (Panels A–D). A minimum of 10 fields for each kidney slide were examined and assigned for severity of changes. The scoring system was as follows: 0, absent; 1, mininal changes; 2, moderate changes and 3, marked changes. Each bar represents mean ± S.E.M. +: P<0.05 *vs*. IR+Saline, *: P<0.05 *vs*.Sham+Saline (N = 6–10 rats).

I/R injury caused a marked increase in total histological score in comparasion with sham-operated animals (IR + Saline *vs*. Sham + Saline; P<0.01). Chloroquine (0.3 mg/kg i.v., administered at three different times; 3 mg/kg i.v., 30 I) reduced tubular necrosis score in comparasion with control group (IR + H03-30I, IR + H03-30R, IR + H03-5R and IR + H3-30I *vs*. IR + Saline; P<0.05) (Fig. 3A). Time-related differences between three administrations of chloroquine were not observed (IR + H03-30I and IR + H03-30R *vs*. IR + H03-5R; P>0.05, all) (Fig. 3B-D).

**Figure 4:**
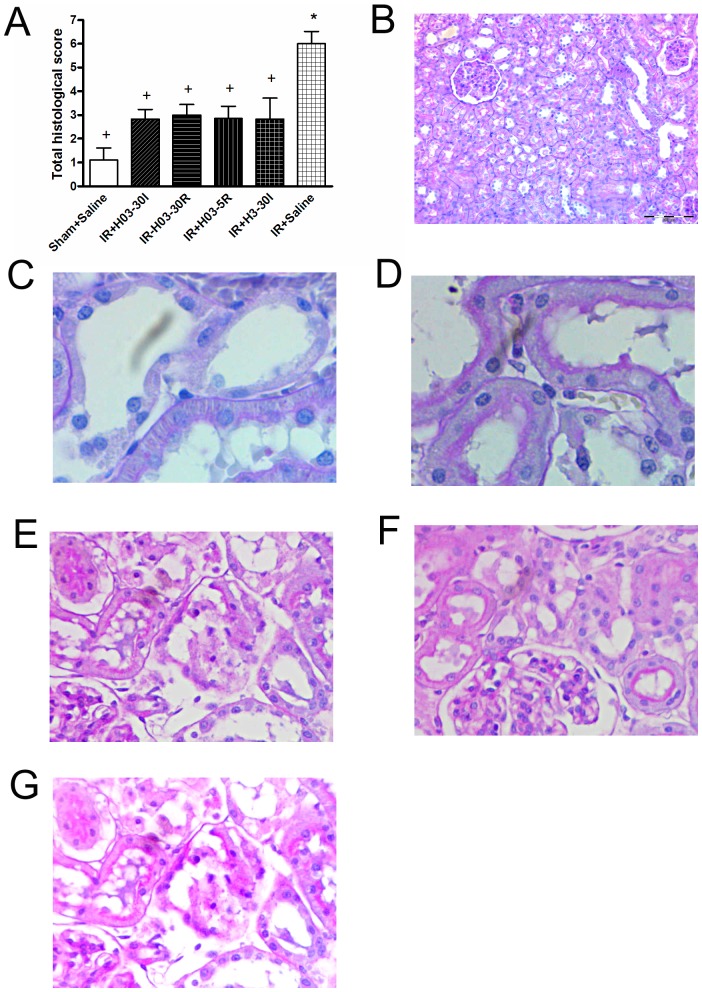
The effects of chloroquine (0.3 mg/kg; 3 mg/kg, i.v. bolus) on total histological score of renal I/R injury and histological micrographs of renal tissues. Chloroquine in dose of 0.3/kg, i.v. was injected 30 min before ischemia (IR+H03-30I), 30 min before reperfusion (IR+ H03-30R) and 5 min before reperfusion (IR+H03-5R), and in dose of 3 mg/kg, i.v. was injected 30 min before ischemia (IR+H3-30I). Control groups, Sham+Saline and IR+Saline received an i.v. bolus of 0.5 ml saline only. A minimum of 10 fields for each kidney slide were examined and assigned for severity of changes. The scoring system was as follows: 0, absent; 1, mininal changes; 2, moderate changes and 3, marked changes. Total histological score was calculated by addition of all scores (Panel A). Each bar represants mean ± S.E.M. +: P<0.05 *vs*. IR+Saline, *: P<0.05 *vs*.Sham+Saline (N = 6–10 rats). Histological micrographs of renal tissues: kidney sections taken from Sham-operated rats or rats subjected to renal I/R injury. Periodic acid–Schiff (PAS) stain coloring. Original magnification ×20. Figures were randomly chosen from the series of at least 6 experiments (Panels B–G). Panel B: Sham-operated animals treated with saline only (Sham+Saline group) - normal renal parenchyma (PAS staining). Panel C: Rats subjected to renal I /R injury, pretreated with chloroquine at 0.3 mg/kg, i.v. 30 min before ischemia (IR+H03-30I group) - moderate kidney damage, about half of proximal tubules show loss of brush border, dilatation of lumen and loss of nuclei in some epithelial cells. Panel D: Rats subjected to renal I /R injury, pretreated with chloroquine at 0.3 mg/kg, i.v. 30 min before reperfusion (IR+ H03-30R group) - moderate kidney damage, loss of brush border was observed in half of proximal tubules, in addition to dilatation of lumen and loss of nuclei in some epithelial cells. Panel E: Rats subjected to renal. I /R injury, pretreated with chloroquine at 0.3 mg/kg, i.v. 5 min before reperfusion (IR+H03-5R group) - moderate kidney damage, two thirds of proximal tubules show loss of brush border, dilatation of lumen and loss of nuclei in numerous epithelial cells. Panel F: Rats subjected to renal. I /R injury, pretreated with chloroquine at 3 mg/kg, i.v. 30 min before ischemia (IR+H3-30I group) - moderate kidney damage, two thirds of proximal tubules show loss of brush border, dilatation of lumen and loss of nuclei in majority of epithelial cells (marked necrosis). Panel G: Rats subjected to renal I / R injury, pretreated with saline only (IR+Saline-group) - marked kidney damage, interstitial edema diffusely present, proximal tubules show loss of brush border and lumen dilatation and loss of nuclei in some epithelial cells.

Also, both doses of chloroquine significantly reduced total histological score (IR + H03-30I, IR + H03-30R, IR + H03-5R and IR + H3-30I *vs*. IR + Saline; P<0.05) (Fig. 4A). Representative light photomicrographs of a kidney section taken from rats colored with PAS are shown in Fig. 4B-G.

**Figure 5:**
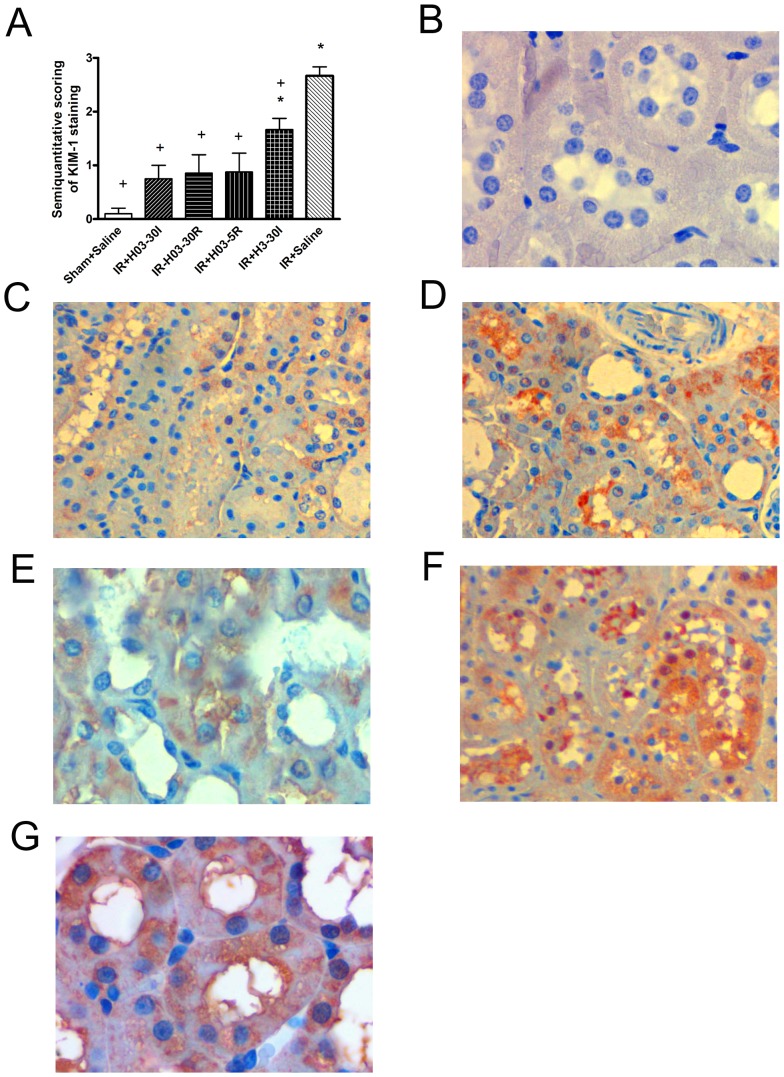
The effects of chloroquine (0.3 mg/kg, i.v; 3 mg/kg, i.v.) on KIM-1 staining score of renal I/R injury and histological micrographs of renal tissues. Chloroquine in dose of 0.3/kg, i.v. was injected 30 min before ischemia (IR+H03-30I), 30 min before reperfusion (IR+ H03-30R) and 5 min before reperfusion (IR+H03-5R), and in dose of 3 mg/kg, i.v. was injected 30 min before ischemia (IR+H3-30I). Control groups, Sham+Saline and IR+ Saline received instead of drug i.v. bolus of 0.5 ml saline only. The following scoring system was used: 0, no staining; 0.5+, focal weak fine granular staining; 1+, weak fine granular staining; 2+, moderate granular staining; and 3+, strong large granular staining. The KIM-1 staining intensity score for each sample was graded by two experts in the field of pathology who was blinded to the group assessments. Finally, KIM-1 staining density score for each sample calculated using mean score of these two experts (mean score ± S.E.M.) (Panel A). Each bar represants mean ± S.E.M. +: P<0.05 *vs*. IR+Saline, *: P<0.05 *vs*.Sham+Saline (N = 6–10 rats). Histological micrographs of renal tissues: kidney sections taken from Sham-operated rats or rats subjected to renal I/R injury. Kidney injury molecul-1 (KIM-1) staining. Original magnification ×20. Figures were randomly chosen from the series of at least 6 experiments (Panels B–G). Panel B: Sham-operated animals treated with saline only (Sham+Saline group) - absence of immunoreactivity for KIM-1. Panel C: Rats subjected to renal I /R injury, pretreated with chloroquine at 0.3 mg/kg, i.v. 30 min before ischemia (IR+H03-30I group) – most of proximal and some distal tubules show mild staining for KIM-1. Panel D: Rats subjected to renal I /R injury, pretreated with chloroquine at 0.3 mg/kg, i.v. 30 min before reperfusion (IR+ H03-30R group) - nearly half of proximal tubules and rare distal tubules show mild to moderate KIM staining.Panel E: Rats subjected to renal I /R injury, pretreated with chloroquine at 0.3 mg/kg, i.v. 5 min before reperfusion (IR+H03-5R group) - nearly half of proximal tubules and rare distal tubules show moderate KIM staining. Panel F: Rats subjected to renal I /R injury, pretreated with chloroquine at 3 mg/kg, i.v. 30 min before ischemia (IR+H3-30I group) - most of proximal and some distal tubules show moderate staining for KIM-1. Panel G: Rats subjected to renal I / R injury, pretreated with saline only (IR+Saline-group) - proximal and distal tubules show moderate to intensive positive KIM staining.

I/R injury caused a marked increase in KIM-1 staining in comparasion with sham-operated animals (IR + Saline *vs*. Sham + Saline; P<0.01). Chloroquine in dose of 0.3 mg/kg i.v. significantly reduced the KIM-1 score when compared to IR + Saline group (IR + H03-30I vs. IR + Saline; P<0.01), while dose of 3 mg/kg i.v. also reduced, but not completely abolished I/R-caused renal injury (IR + H3-30I vs. Sham + Saline; P<0.05). (Fig. 5A).

Representative light photomicrographs of kidney section taken from rats subjected to immunohistochemical staining for KIM-1 are shown at Fig. 5B-G.

In our study, we checked correlations between both biochemical and histological parametars of I/R injury and KIM-1 staining in all subjected groups. We found highly positive correlation of KIM-1 staining scores with sCre (r = 0,8463; p<0,0001; Cl 0,6874 to 0,9279) and total histological score (r = 0,8093; p<0,0001; Cl 0,5884 to 0,9178). KIM-1 expression was also positively associated with proximal tubular necrosis score (r = 0,6538; p<0,001; Cl 0,3204 to 0,8431), cast formation score (r = 0,6405; p<0,001; Cl 0,2998 to 0,8363) and sUr (r = 0,6422; p<0,001; Cl 0,3467 to 0,8217).

## Discussion

I/R injury that often leads to AKI still remains an unresolved problem in pharmacotherapy. Acute tubular necrosis due to I/R injury in patients is associated with a high mortality rate and there has not been significant progress in therapy in the past few decades [24]–[27]. In this paper, we have shown that pretreatment with chloroquine (0.3 mg/kg, i.v. bolus) causes substantial reduction in biochemical and histological parametars and, also, in KIM-1 staining score.

Chloroquine, well-known antimalarial drug, possesses some other, so called pleitropic effects: antiinflammatory, anticoagulant and vascular actions. Also, there is evidence that chloroquine treatment could effects antioxidant enzymes and has a favorable effects on serum lipid profile and glucose concentrations. It seems that these effects of chloroquine are dose-dependant [28]–[35].

In our study we examined chloroquine pretreatment at doses of 0.3 mg/kg, 3 mg/kg and 30 mg/kg (i.v., bolus) 30 min before ischemia, 30 min before reperfusion and 5 min before reperfusion. Time-related differences between pretreatments were not observed in either of groups. Of note, dose-range interval for chloroquine treatment in humans is similar (it goes between 2.5–25 mg/kg daily) [36]. A dose of 30 mg/kg of chloroquine per se caused a marked decrease in the mean arterial pressure during the i.v. application, which could be explained by vasodilatation and cardiac depression in rats [37]. So, in this paper we have discussed the effects of the lower doses of chloroquine.

We showed for the first time that chloroquine in the lowest dose (0.3 mg/kg, i.v.) significantly reduced some biochemical (sUr, sCre, FENa^+^), histological parametars (total histological score, tubular necrosis score) and attenuates KIM-1 staining score. It should be pointed out that chloroquine in dose of 3 mg/kg has also shown some protective effects, but not completely abolished renal I/R injury in rats.

Kidney injury molecule-1 (KIM-1) is a type 1 transmembrane glycoprotein which is normally minimally expressed in kidney tissue or urine [38]–[40]. It is believed that KIM-1 plays role in the process of both kidney injury and healing, but precise mechanism of KIM-1 restoration of tubular integrity after injury is not fully understood. In the past 15 years (1998–2013) Edelstein and collaborators have published several papers on the importance and clinical applicability of new biomarkers of acute renal failure. Recently, his team has demonstrated that in ischemic injury KIM-1 expression is most prominent in the S3 segment (i.e., the segment most susceptible to ischemic injury) [41].

In our experimental model, first changes in standard biomarkers of acute renal failure due to I/R injury could be detected within two hours after the begining of reperfusion [14]. After four hours of reperfusion, there is clear and reliable increase in serum creatinine, urea and fractional excretion of sodium due to I/R injury (Fig. 2). Moreover, the protective effect of a single i.v. dose of chloroquine (0.3 mg/kg i.v.) on kidney function and morphology was shown using the same biomarkers. KIM-1 expression is more sensitive than normal histology for detection of low-grade proximal tubule injury [42]. In the present experiments KIM-1 changes over time were not assessed.

Many preclinical animal studies showed that urinary KIM-1 was early biomarkers of AKI in mice and rats (animals were underwent to ischemic injury lasting 10 min or longer). Changes in urinary KIM-1 correlated with KIM-1 immunostaining in the proximal tubular epithelial cells [43]–[46].

There are several possible explanations of our findings, and both pharmacodynamic and pharmacokinetic factors should be taken in consideration.

First of all, pharmacokinetic profile of chloroquine allows us to suppose that it easily reaches the kidney [47]. Current evidence suggests that chloroquine may affect kidney function administered either acutely or chronically in rats, probably due to its accumulation in kidney cells [48], [49]. The deposition of chloroquine in the epithelial tubular cells may alter perfusion pressure of the kidney and renal haemodynamics, also affecting renal fluid and electrolyte handling. It is shown that it can stimulate significant increase in urine flow rate, glomerular filtration rate and sodium excretion [9].

The explanation of chloroquine pharmacodynamics in the present model of I/R injury should take into consideration its effect on endothelial NO production. The effects of chloroquine on renal function probably involves stimulation of nitric oxide synthase [11], [50]. The stimulatory effects of chloroquine on NO production were also demonstrated in mouse, pig and human endothelial cells *in vitro* [51]. Enhancements of the NO level by chloroquine potentiates vasodilatation of resistant vessels, leading to preservation of tissue perfusion after I/R injury.

Nevertheless, we have already discussed the antiinflammatory effects of chloroquine. It was shown that it decreases the production of some proinflammatory cytokines, but also exerts antiinflammatory effects via non-lysosomotropic mechanism [27], [52].

Synthesis of proinflammatory cytokines such as interleukin (IL)-1, IL-6 and tumor necrosis factor (TNF)-α, increasing in cell adhesion-molecule expression, development of oxidative stress are just some of the changes that occur during acute kidney injury. It is shown that chloroquine treatment inhibits production of IL-1 and IL-6 in human monocytes and T cells [53]. Some recent studies show that chloroquine administration attenuates the decline in renal function and production of typical serum pro- and anti-inflammatory cytokines TNF-α and IL-10 [54]. Other authors confirmed that chloroquine exerts some anti-inflammatory effects through the down-regulation of (TNF)-α production and signaling in macrophages, as well, as the cytokine pattern production [55].

Also, chloroquine, as a cationic drug, accumulates in acidic cellular compartments and binds to phospholids with a consequent increase in lysosomal pH and induce phospholipidosis. It seems that impairement of lysosomal function by chloroquine leads to anti-inflammatory effects by inhibition of arachidonic acid release and prostaglandin E2 synthesis [56]. Cooper and colleagues considered that lysosomotropic effects of chloroquine are widely responsible for its anti-inflammatory properties (decreasing in production of proinflammatory cytokines such as: IFN-γ, TNF-α, IL-1, IL-6), but also emphasized the importance of non-lysosomotropic mechanisms (it was shown that chloroquine could inhibit TNF-α release in macrophages through inhibition of TNF-α mRNA synthesis) [10].

Despite this, chloroquine is well-known as autophagy inhibitor. Autophagy is induced in renal tubular cells during acute kidney injury; it was not obvious during the ischemia period, but was significantly enhanced during reperfusion. Some authors considered that autophagic effects of chloroquine are concentration dependent and that would be critical to measure actual concentrations, not only in blood, but also in tissue [57]. However, whether this autophagy during I/R injury is protective or injurious remains controversial. Some increasing evidence suggests that autophagy may provide a protective mechanism for cell survival [58]–[61]. On the other hand, some authors describe dual role of chloroquine in liver I/R injury: reduction of damage in early phase (0–6 h after reperfusion), but aggraviation in late phase (24–48 h after reperfusion). The mechanism of protection involving modulation of mitogen-activated protein kinase activation and inflammatory cytokines production, whereas chloroquine worsened liver injury *via* inhibition of autophagy [62].

Finally, we should not rule out other potentially protective mechanisms of chloroquine in I/R injury such as inhibition of stimulation by Toll-like receptors and prostaglandin synthesis, effects on PARP and PPAR receptors etc. [51].

## Conclusion

Our study shows for the first time that a single dose of chloroquine (0.3 mg/kg i.v.) could afford significant protection of the injured rat kidney. It is important to notice that protective effects of chloroquine in the present experimental model of I/R injury is not time-dependant, and the drug could be administered immediately before reperfusion.

Beneficial effects of acute pretreatment with a single dose of chloroquine in the rat model of renal I/R injury could be confirmed by KIM-1 staining scores. Our study provides the first evidence that KIM-1 staining scores could be used as an indicator of the therapeutic benefit of different pharmacological agents in the experimental model of renal I/R injury. KIM-1 reliably confirmed that chloroquine affords an acute protective effect on kidney function and morphology.

Further experiments are needed to assess whether chemical modifications of chloroquine molecule may influence its pharmacodynamics and/or pharmacokinetics in the present experimental model.
